# Neuroprotective Effects of Germinated Brown Rice against Hydrogen Peroxide Induced Cell Death in Human SH-SY5Y Cells

**DOI:** 10.3390/ijms13089692

**Published:** 2012-08-03

**Authors:** Norsharina Ismail, Maznah Ismail, Siti Farhana Fathy, Siti Nor Asma Musa, Mustapha Umar Imam, Jhi Biau Foo, Shahid Iqbal

**Affiliations:** 1 Nutricosmeceuticals and Nutrigenomics Programme, Laboratory of Molecular Biomedicine, Institute of Bioscience, Universiti Putra Malaysia, Serdang, Selangor 43400, Malaysia; E-Mails: maznah@medic.upm.edu.my (M.I.); farhana811@hotmail.com (S.F.F.); ana_insyirah85@yahoo.co.uk (S.N.A.M.); mustyimam@gmail.com (M.U.I.); foojhibiau@gmail.com (J.B.F.); ranashahid313@gmail.com (S.I.); 2 Department of Nutrition and Dietetics, Faculty of Medicine and Health Sciences, Universiti Putra Malaysia, Serdang, Selangor 43400, Malaysia; 3 Department of Chemistry, University of Sargodha, Sargodha 40100, Pakistan

**Keywords:** germinated brown rice, γ-aminobutyric acid, hydrogen peroxide, neuroprotective, SH-SY5Y cell death

## Abstract

The neuroprotective and antioxidative effects of germinated brown rice (GBR), brown rice (BR) and commercially available γ-aminobutyric acid (GABA) against cell death induced by hydrogen peroxide (H_2_O_2_) in human neuroblastoma SH-SY5Y cells have been investigated. Results show that GBR suppressed H_2_O_2_-mediated cytotoxicity and induced G0/G1 phase cell cycle arrest in SH-SY5Y cells. Moreover, GBR reduced mitochondrial membrane potential **(**MMP) and prevented phosphatidylserine (PS) translocation in SH-SY5Y cells, key features of apoptosis, and subsequent cell death. GBR exhibited better neuroprotective and antioxidative activities as compared to BR and GABA. These results indicate that GBR possesses high antioxidative activities and suppressed cell death in SH-SY5Y cells by blocking the cell cycle re-entry and apoptotic mechanisms. Therefore, GBR could be developed as a value added functional food to prevent neurodegenerative diseases caused by oxidative stress and apoptosis.

## 1. Introduction

Oxidative stress is implicated in the neuronal damage associated with Alzheimer’s disease, Parkinson’s disease, Huntington’s disease, amyotropic lateral sclerosis and cerebral ischemic stroke. The damage is thought to be mediated by reactive oxygen species (ROS) including hydrogen peroxide (H_2_O_2_). The human neuroblastoma SH-SY5Y cell line is widely used as a model cell system for studying oxidative stress-induced neuronal cell death. SY-SY5Y cells can be differentiated using retinoic acid (RA) with characteristics similar to neurons [1].

H_2_O_2_-induced cell death involves apoptosis and necrosis in a concentration dependent manner in SH-SY5Y cells, as shown by flow cytometric analysis of Annexin V-fluorescein isothiocyanate (FITC) and propidium iodide staining, PS translocation [2], decrease in SH-SY5Y cell viability and mitochondrial membrane depolarization [3]. In addition to this, cell cycle is a highly regulated process with numerous checkpoints to ensure homeostatic balance between cell proliferation and cell death under appropriate environmental signals, thus the defective cells are efficiently removed during cell cycle process [4]. In terminally differentiated neurons, cells can exit the cell cycle and stay at resting (G0) phase [5]. Nevertheless, induction of oxidative stress by factors such as H_2_O_2_ could lead to aberrant cell cycle re-entry into G0/G1 phase through several pathways.

Recently, physicians, health professionals and researchers have focused attention towards natural substances rather than synthetic chemicals with neuroprotective potential that can scavenge free radicals and protect cells from oxidative damage [3]. Germination of grains is one way of enhancing their nutritional value as proven in the case of germinated wheat, soybean seeds [6,7] and brown rice (BR) [8]. Specifically, germination of BR is driven by the need to reduce its chewiness, though in the process major bioactive compounds have been shown to increase in concentration. When compared to milled rice, germination has been proven to increase γ-aminobutyric acid (GABA) by 10-fold, and dietary fiber, vitamin E, niacin and lysine by nearly 4-fold. In addition, vitamins B_1_ and B_6_, and magnesium are increased by nearly 3-fold [9]. Germination also softens the hard texture of BR and makes grain nutrients easier to digest and absorb [10,11].

It has been reported that GBR intake may ameliorate oxidative stress related diabetic vascular complications such as retinopathy and nephropathy [12]. In addition, GBR reduces cholesterol level [13] and risks of cardiovascular disease. The components of GBR that possibly contribute in improvement of lipid profile include monounsaturated fatty acid, GABA, tocopherol, and γ-oryzanol [14]. On the other hand, it is shown that feeding on GBR diet decreased the accumulation of lead and hence improved learning and memory deficits in developing rats after Pb exposure that may be due to the antioxidative effects of phenolic compounds and high content of GABA [15]. GBR has also been shown to possess antidepressant effects in mice [16]. Another report has shown that GBR was able to improve learning ability in Aβ-induced learning and memory deficits in mice, likely as a result of high amounts of GABA in GBR, through modulation of glutamatergic system resulting in memory enhancement [17]. Specifically, water extract of GBR on neuroblastoma SK-N-SH cells was shown to protect against H_2_O_2_-induced cytotoxicity [18]. Also, phenolic-rich fraction of 80% ethanolic extract of *Schisandra chinensis* fruits was protective against H_2_O_2_-induced cytotoxicity in SH-SY5Y cells [19] underscoring our choice of ethanolic extract for the current study.

Nevertheless, studies on the direct effects of GBR on H_2_O_2_-induced cytotoxicity and apoptosis are limited, and since neurodegeneration due to oxidative stress is believed to underlie the neurodegenerative diseases, it may be hypothesized that GBR, BR and GABA have some neuroprotective effects. The present study was aimed to examine the neuroprotective and antioxidative effects of GBR in comparison to BR and GABA against H_2_O_2_ in RA-differentiated SH-SY5Y neuronal-like cells.

## 2. Results and Discussion

### 2.1. GABA, GBR and BR Preserved SH-SY5Y Cells against H_2_O_2_-Induced Cytotoxicity

**Figure 1:**
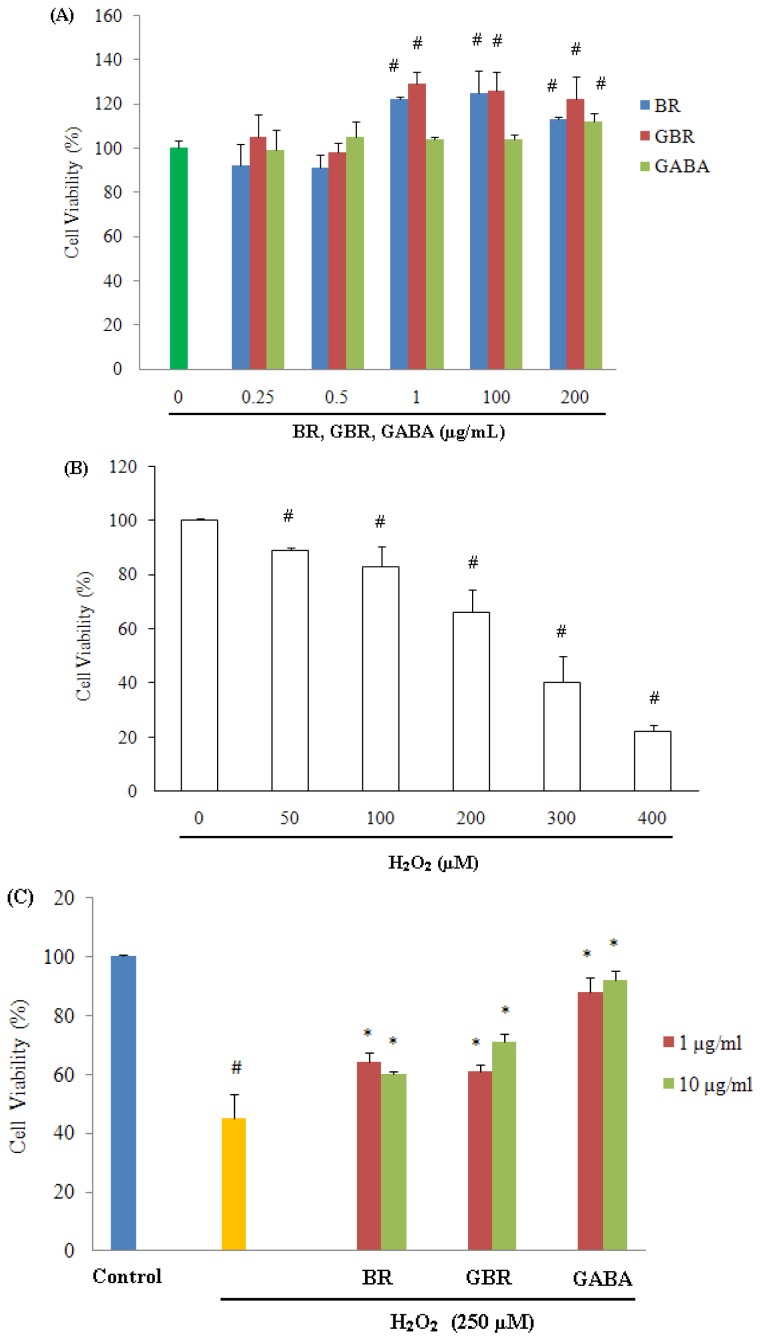
Neuroprotective effects of germinated brown rice (GBR), brown rice (BR) and γ-aminobutyric acid (GABA) on H_2_O_2_-induced cytotoxicity in SH-SY5Y cells determined by (3-[4,5-dimethylthiazol-2-yl]-2,5-diphenyl-tetrazolium bromide) (MTT) assay. (**A**) Neurotoxicity effects of GBR, BR and GABA on SH-SY5Y cell viability. Human SH-SY5Y neuroblastoma cells were incubated with GBR, BR or GABA alone at 0.25–200 μg/mL for 24 h; (**B**) The neurotoxicity of H_2_O_2_ on SH-SY5Y cells. SH-SY5Y cells were treated with 50–400 μM H_2_O_2_ for 24 h; (**C**) SH-SY5Y cells were pretreated with 1 and 10 μg/mL of GBR, BR, GABA or DMSO diluted in serum-free medium for 24 and then incubated with or without H_2_O_2_ (250 μM) for an additional 24 h. Results are the mean ± SD in triplicates. * *p* < 0.01 *versus* H_2_O_2_, # *p* < 0.01 *versus* control.

Survival of SH-SY5Y cells exposed to H_2_O_2_ in the absence and presence of GABA, GBR and BR was evaluated using (3-[4,5-dimethylthiazol-2-yl]-2,5-diphenyl-tetrazolium bromide) (MTT) assay. Exposure of SH-SY5Y cells to GABA, GBR and BR separately, up to 24 h over concentration range of 1–200 μg/mL, produced no significant alteration in cell viability, rather it stimulated the growth of cells as compared to untreated control (Figure 1A). On the other hand, exposure of cells to 250 μM H_2_O_2_ for 24 h resulted in approximately 50% cell cytotoxicity in comparison to control cells (*p* < 0.01) (Figure 1B). Therefore, 250 μM H_2_O_2_ was chosen for incubation of SH-SY5Y cells for 24 h to induce cell death in all subsequent experiments.

In contrast, pre-treatment with 1 or 10 μg/mL of GABA, GBR and BR significantly increased the viability of SH-SY5Y cells against H_2_O_2_-induced cytotoxicity (Figure 1C). At 10 μg/mL concentration, cell viability was found to be 92% ± 3%, 71% ± 2.7% and 60% ± 1% for GABA, GBR and BR respectively. At the lowest tested concentration, *i.e.*, 1 μg/mL, GABA, GBR and BR were able to maintain cell viability at 88% ± 5%, 61% ± 2.3% and 64% ± 3.2% respectively (Figure 1C). H_2_O_2_ has the ability to induce cell cytotoxicity [20] and results of the current study demonstrated that the physiological concentration of 1 and 10 μg/mL of GABA, GBR and BR were able to counteract the deleterious effect of 250 μM H_2_O_2_ (Figure 1C).

Furthermore, aqueous extract of GBR showed no toxicity on human neuroblastoma SK-N-SH cells up to 4000 μg/mL for 24 h. Pre-treatment of the cells with aqueous extract of GBR at 2000 μg/mL followed by exposure to 150 μM H_2_O_2_ protected the cells from H_2_O_2_-induced cytotoxicity [18]. In this study, protective effect of GBR ethanolic extract was examined against H_2_O_2_-induced cytotoxicity at lower concentrations up to 200 μg/mL. Phenolic-rich fraction obtained from 80% ethanolic extract of *Schisandra chinensis* fruits protected SH-SY5Y cells against H_2_O_2_-induced cytotoxicity [19], which is in agreement with the findings of this study, whereby ethanolic extract from natural substances protects neuroblastoma cell lines against H_2_O_2_-induced cytoxicity. Crude plant extracts usually exhibit superior health oriented properties than purified compounds, due to less toxicity and synergistic effects of crude extracts [21].

### 2.2. GBR and GABA Arrested SH-SY5Y Cells at G0/G1 Phase

**Figure 2:**
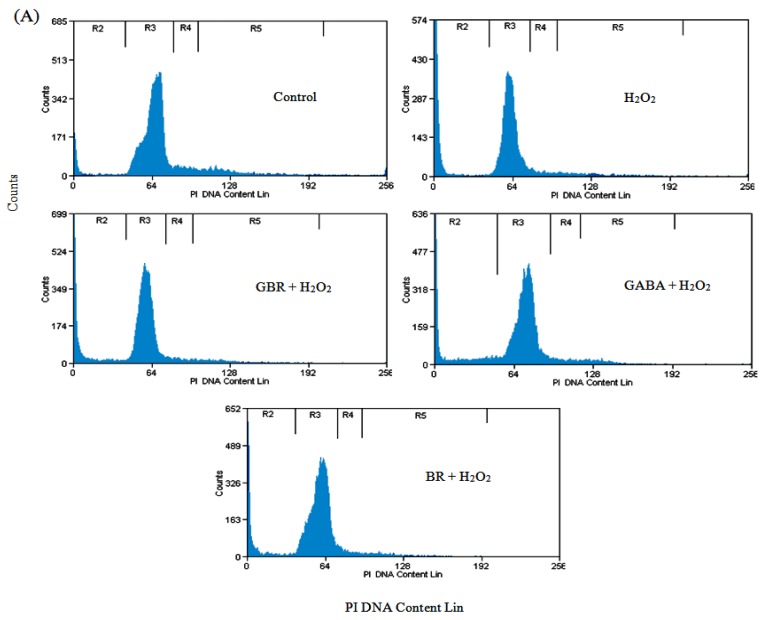

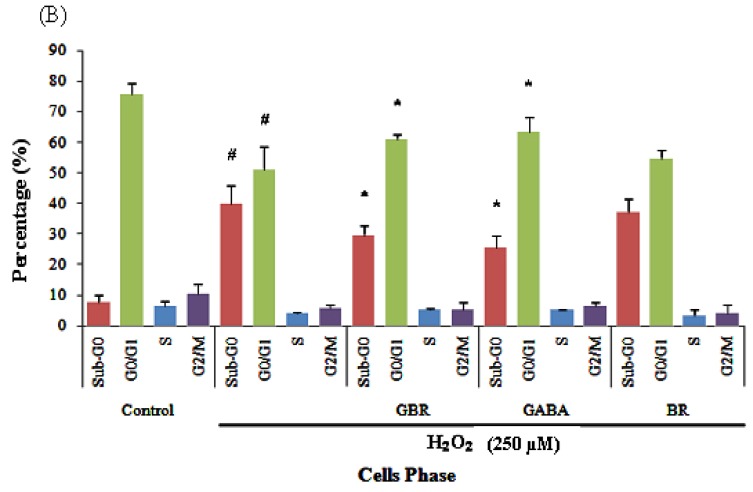
Flow cytometric measurement of cell death and cell cycle on pretreated GBR, GABA and BR (100 μg/mL) exposed to 250 μM H_2_O_2_ over 24 h. (**A**) Histograms on cell death and cell cycle distribution. R2: Sub-G0 population/cell death indicative of DNA damage was analyzed from the hypo diploid fraction (<2n DNA) of DNA cell cycle analysis; R3: G0/G1 population indicative of living cells; R4: S population indicative of cells undergoing synthesis; R5: G2/M indicative of dividing cells; (**B**) Bar diagrams on cell death and cell cycle distribution. Results are the mean ± SD in triplicates. * *p* < 0.01 *versus* H_2_O_2_, # *p* < 0.01 *versus* control.

Cell death and cell cycle population, in terminally differentiated SH-SY5Y cells exposed to H_2_O_2_, was determined in the absence or presence of GBR, GABA and BR by flow cytometric analysis using propidium iodide staining. The results showed significant induction of dead cell population (8% ± 2.34% to 40% ± 5.88%) indicated as Sub-G0 upon exposure to 250 μM H_2_O_2_ in comparison to control cells (Figure 2(A,B)). The presence of 250 μM H_2_O_2_ may cause DNA or protein damage, chromatin destruction and manipulation of a quiescent G0 terminally differentiated neuron back into the cell cycle [22]. Quiescent cells reenter the cell cycle to repair the damages caused due to H_2_O_2_ insults or initiate cell death if the damage is too extensive to be repaired [23]. Oxidative stress has been suggested as the mechanism responsible for cell cycle reentry as seen with studies performed on neuronal cell preparations [24].

Conversely, cells pretreated with 100 μg/mL of GBR, GABA and BR showed reduction in dead cells to 30% ± 2.99%, 26% ± 3.92% and 37% ± 4.32%, respectively (Figure 2(A,B)). GBR and GABA were found to be better at protecting the cells against H_2_O_2_-induced toxicity as compared to BR. Meanwhile, the increment of cell cycle G0/G1 phase activity with GBR and GABA pretreatment, compared to H_2_O_2_ alone, indicated that the H_2_O_2_-treated cells were arrested at G0/G1 phase with an increased activity at Sub-G0 phase (Figure 2(A,B)). It is suggested that lower concentrations of H_2_O_2_ cause repairable DNA double strand breaks (DSBs) and activation of G0/G1 components of cell cycle machinery [25], which is indicated by an increased activity of G0/G1 cell cycle components on flow cytometric analysis. However, higher concentrations of H_2_O_2_ induce major DSBs in neurons leading to cell death [25], which is indicated by increased activity of Sub-G0 cell cycle components.

From our data, it can be seen that there was no significant difference in cell populations in S and G2/M phases among pretreatments with BR, GBR and GABA against H_2_O_2_. Activity at Sub-G0 phase indicating cell death was highest for H_2_O_2_ suggesting GBR and GABA had protective effects against cell death better than BR. This data supports our neurotoxicity data for BR pretreatment when compared to GBR and GABA.

### 2.3. GBR and GABA Prevented H_2_O_2_-Induced Reduction of Mitochondrial Membrane Potential (MMP) in SH-SY5Y Cells

**Figure 3:**
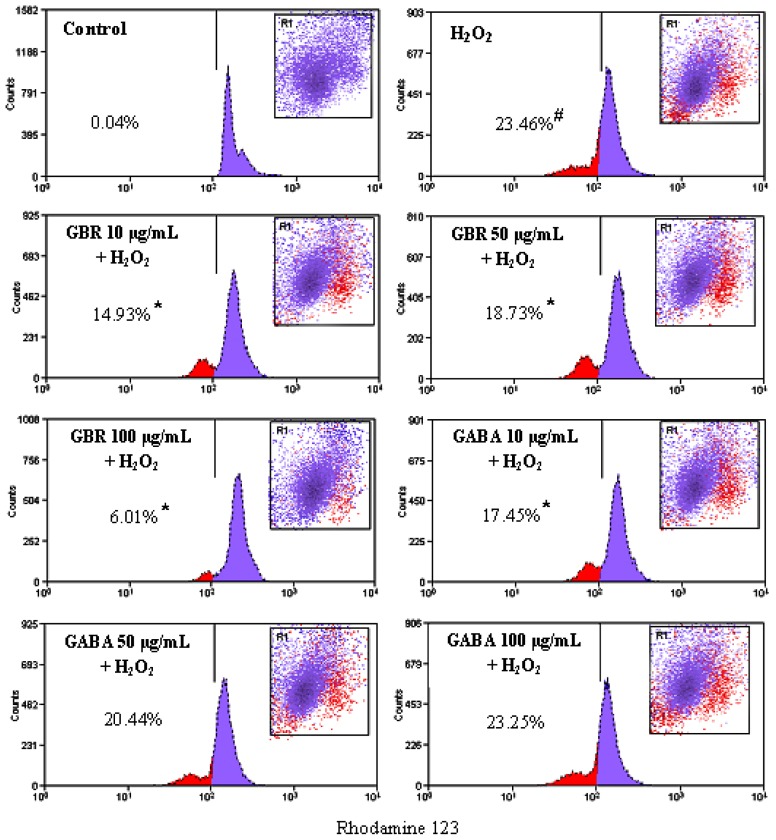
Effects of GBR and GABA on H_2_O_2_-induced reduction of mitochondrial membrane potential. Cells were pre-treated with 10, 50 and 100 μg/mL of GBR and GABA, respectively followed by 250 μM H_2_O_2_ challenge for 24 h. After incubation, cells were stained with Rhodamine 123 and analyzed by flow cytometer. The reduced fluorescence of Rhodamine 123 was determined as the reduced mitochondrial membrane potential. Results are the mean ± SD in triplicates. * *p* < 0.01 *versus* H_2_O_2_, # *p* < 0.01 *versus* control.

Collapse of MMP event provides an early indication of the initiation of cellular apoptosis. MMP was found to be reduced rapidly, when SH-SY5Y cells were exposed to 250 μM H_2_O_2_ for 24 h, which was detected by weakening the fluorescence intensity of a mitochondrial fluorescence probe, Rhodamine 123. As compared to control cells, H_2_O_2_ treatment increased the Rhodamine 123 negative cells from 0.04 to 23.46%. Pretreatment with 10, 50 and 100 μg/mL of GBR protected cells against H_2_O_2_-induced lowering of MMP, decreasing Rhodamine negative cells to 14.93%, 18.73% and 6.01%, respectively. Meanwhile, pretreatment with 10, 50 and 100 μg/mL of GABA weakly protected the cells against H_2_O_2_-induced lowering of MMP, decreasing Rhodamine negative cells to 17.45, 20.44 and 23.25% respectively (Figure 3).

H_2_O_2_ exposure is known to induce mitochondrial membrane depolarization in SH-SY5Y cells [26]. Inhibition of intracellular ROS accumulation has been linked to reduction of mitochodrial membrane depolarization [26] while antioxidants are known to prevent loss of MMP [27]. Our findings showed that GBR prevented H_2_O_2_-induced mitochondrial membrane depolarization, which may be due to inhibition of ROS accumulation as found previously, where GBR inhibited H_2_O_2_-induced ROS generation in neuronal SK-N-SH cells [18].

Exposure of SH-SY5Y cells to H_2_O_2_ caused a rapid increase in ROS production [27]. Pretreatment of SH-SY5Y cells with plant bioactives, prior to exposure to H_2_O_2_, has been shown to significantly increase levels of superoxide dismutase (SOD), which is an intracellular antioxidant enzyme, suggesting that addition of exogenous bioactive to the cells may activate internal antioxidant enzymes to protect the cells against ROS [27]. In the present study, the antioxidative properties of phenolic compounds, in exogenously added GBR to SH-SY5Y cells, may have activated SOD to inhibit H_2_O_2_-induced accumulation of ROS. GBR was found to be better at preventing H_2_O_2_-induced reduction of MMP in comparison to GABA, likely as a result of its higher antioxidative properties. Despite showing lower cell viability, it is likely that GBR produced better MMP due to activation of certain protective mechanisms within the mitochondria as hypothesized in mitohormesis, hence the enhanced MMP compared to GABA. Interestingly, mitohormesis has recently been linked to longer life span, and our data suggests that GBR may confer a superior protective effect compared to GABA on a long term. Mitochondrial protective effects of GBR may then inhibit the release of pro-apoptotic components from the mitochondria.

### 2.4. GBR Prevented H_2_O_2_-Induced Apoptosis and Necrosis in SH-SY5Y Cells

**Figure 4:**
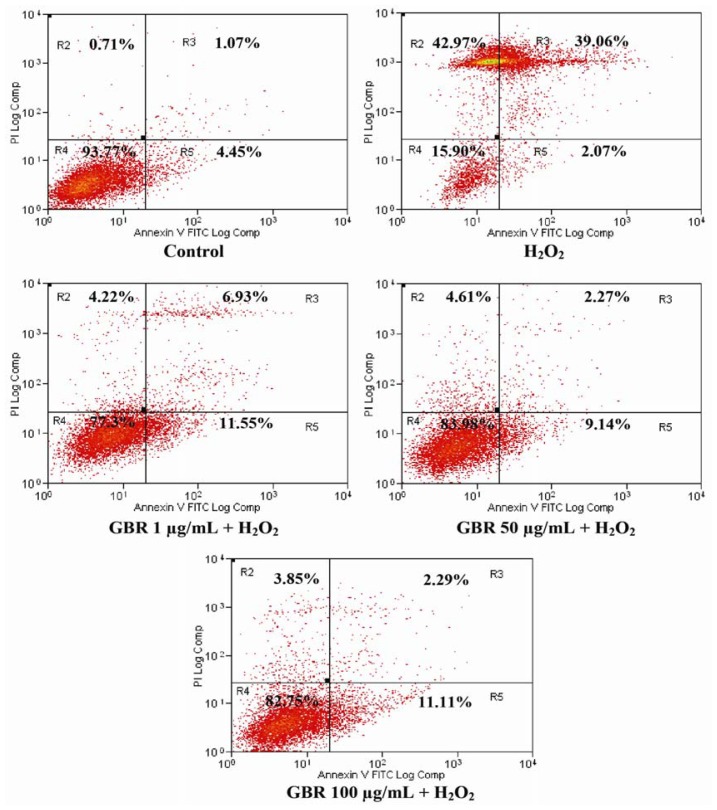
Flow cytometric determination of apoptotic and necrotic cell death after exposure of SH-SY5Y cells to 250 μM H_2_O_2_ as measured by Annexin V-FITC staining assay. Control, untreated SH-SY5Y cells; SH-SY5Y cells treated with 250 μM H_2_O_2_ for 24 h; exposure of SH-SY5Y cells to 250 μM H_2_O_2_ over 24 h in the presence or absence of 24 h pre-treated 1, 50 and 100 μg/mL GBR. R2: late necrosis; R3: late apoptosis/early necrosis; R4: viable cells; R5: early apoptosis. Results are the mean ± SD in triplicates. * *p* < 0.01 *versus* H_2_O_2_, # *p* < 0.01 *versus* control.

During apoptosis, PS is translocated from inner side of the plasma membrane to the outer layer for FITC binding. In this study, the exposure of SH-SY5Y cells to H_2_O_2_ showed significant differences in viable (R4), early apoptosis (R5), late apoptosis/early necrosis (R3) and late necrosis (R2) states as compared to control (Figure 4). Control showed 94 ± 3.67% of viable cells, while incubation with 250 μM H_2_O_2_ reduced the cell viability to only 16% ± 3.97% with 2% ± 0.05%, 39% ± 2.2% and 43% ± 3.85% showing early apoptosis, late apoptosis and necrosis, respectively. Therefore, this result indicated that H_2_O_2_ at 250 μM was toxic to SH-SY5Y cells. However, pretreatment of GBR showed neuroprotective effects against H_2_O_2_-induced apoptosis, and GBR showed 77% ± 4.49%, 84% ± 4.21% and 83% ± 4.13% of viable cells at 1, 50 and 100 μg/mL, respectively (Figure 4).

GBR pretreatment protected the cells from rapidly undergoing death induced by H_2_O_2_ by preventing PS translocation, thus the cell membrane remained intact. Early apoptotic cells indicated that PS translocation had left while the cell membrane was still intact. Exposure of cells to 250 μM H_2_O_2_ induced cell death, as ~82% of cells were at early and late apoptotic, and necrotic stages showing that PS translocation had occurred and the cell membrane was damaged. Apoptosis induction by H_2_O_2_ can be transmitted via receptor activation and cytochrome c release from the mitochondria into the cytoplasm, followed by the breakdown of MMP and then activation of caspases. Consequently, PS translocation to the outer leaflet of the cell membrane is the first evidence of morphological change, which later leads to cell shrinkage, blebbing and DNA fragmentation.

### 2.5. Antioxidant Activities of GBR, BR and GABA

GABA content in GBR and BR were determined by Liquid Chromatography Mass Spectrometry (LCMS). GBR was produced by soaking BR in water to induce slight germination, due to which level of GABA increased (Table 1). To provide insight into the relationship between neuroprotective and the antioxidant effects, the antioxidant effects of GBR, BR and GABA were determined. GBR exhibited the best scavenging ability on 2,2-diphenyl-1-picrylhydrazyl (DPPH) radicals (5.66 mg Trolox eq/g extract), followed by BR and GABA with 4.3 and 1.77 mg Trolox eq/g extract, respectively (Table 1). Results of the 2,2′-azino-bis(3-ethylbenzothiazoline-6-sulphonic acid) (ABTS) radical cation decolorization assay showed that GBR had the highest scavenging effect when compared to BR and GABA (Table 1). Among the extracts, GBR and BR showed no significant differences in ferrous iron chelating ability, which is 4.55 ± 0.09 and 4.26 ± 0.24 mg EDTA equivalent/g extract, respectively. These abilities were significantly higher (*P* < 0.05) than that of GABA (Table 1).

Among the antioxidant assays conducted, GBR had consistently showed the highest antioxidant activity followed by BR and GABA for DPPH radical scavenging, ABTS radical cation scavenging and ferrous ion chelating methods (Table 1). The stable DPPH radical is widely used to evaluate the free radical scavenging activity of hydrogen donating antioxidants in many plant extracts [28]. In addition, ABTS method is also widely employed for measuring the relative radical scavenging activity of hydrogen donating and chain breaking antioxidants in many plants extracts [29]. Results demonstrate consistent results in scavenging activities by GBR, BR and GABA in DPPH and ABTS radical scavenging assays; as both assays follow the same principles.

On the other hand, although iron is essential for oxygen transport, respiration, and activity of certain enzymes, it is a reactive metal that catalyzes oxidative damage in living tissues and cells [30]. Iron (II) chelating properties of an antioxidant extract may be attributed to their endogenous chelating agents, mainly phenolics. Certain phenolic compounds have properly oriented functional groups, which can chelate metal ions [31]. In this study, GBR and BR showed higher activities in ferrous ions chelating ability compared to GABA. The phenolic functional group may partly or partially explain the chelating properties of GBR and BR. However, GBR contains slightly lower amounts of soluble phenolic compounds compared to BR due to approximately 70% decrement of the major soluble phenolic compounds in BR, 6′-*O*-(*E*)-feruloylsucrose and 6′-*O*-(*E*)-sinapoylsucrose during germination [32]. BR contains about 3 times higher polyphenolics as compared to white rice in methanolic extracts. This suggests that the milling process removes significant amounts of polyphenolics. The identified phenolic compounds in GBR and BR include protocatechuic, hydroxybenzoic, vanillic, syringic, chlorogenic, caffeic, *p*-coumaric, ferulic, and sinapinic acids besides 6′-*O*-(*E*)-feruloylsucrose and 6′-*O*-(*E*)-sinapoylsucrose [32].

Previous studies have identified several bioactives in GBR including GABA, phytic acid, ferulic acid, tocotrienols and γ-oryzanol. Phytic acid (IP6) was reported to be protective against 6-hydroxydopamine-(6-OHDA)-induced cell apoptosis in rat mesencephalic dopaminergic cells under both normal and iron-excess conditions, thus IP6 may offer neuroprotection in Parkinson’s disease [33]. On the other hand, ferulic acid attenuated cognitive deficits and increase in carbonyl proteins induced by buthionine-sulfoximine in mice [34]. Papaya fruit skin which contains ferulic acid, *p*-coumaric acid, caffeic acid, carotenoids and vitamin C was reported to protect human cells from oxidative stress [35]. Meanwhile, tocotrienols have been shown to protect the neurite from beading, which is one of the earliest events during neuronal degeneration prior to induction of death in H_2_O_2_-treated neurons [36]. Therefore, the neuroprotective effects of GBR against H_2_O_2_, in this study, may be due to the contributions of these bioactives rather than GABA alone.

## 3. Experimental Section

### 3.1. Materials

Human neuroblastoma SH-SY5Y cell line was obtained from ATCC (Manassas, VA, USA). Minimum essential Eagle’s medium, Ham’s nutrient mixture F-12, fetal bovine serum, gentamicin, 2,2-diphenyl-1-picrylhydrazyl (DPPH) radical and all other chemicals were obtained from Sigma (St. Louis, MO, USA).

### 3.2. Production of GBR

Brown rice (2 kg) was rinsed 5 times with tap water, out of which 500 g was soaked in sodium hypocloride (19 mL; 5.25%) solution for 30 min followed by washing with tap water for at least 1 min and soaking in H_2_O_2_ (20 mL; 25%) solution for 6 h at 35 °C in an incubator. After filtration, obtained BR sample was placed in a closed container (15 × 8 × 4 cm) at 35 °C for 18 h. About 10% of the total sample was removed for estimating the efficiency of germination. Rest of the GBR was transferred onto a tray and was kept in an oven at 50 °C, until the moisture content reached 8%–13%. Weight of dried GBR was recorded, and samples were packed in zipped bags and stored in chiller till further analyses.

### 3.3. Extraction of GBR and BR

Ground samples (100 g each of GBR and BR) were homogenized with ethanol (400 mL; 70%) for 30 min and centrifuged at 34,800 *g* for 20 min. The supernatant was filtered through Whattman No. 1 filter paper. The process was repeated twice further to ensure maximum extraction, supernatant of all the three batches was pooled and dried using rotatory evaporator (Rotavapor^®^ R-210, BUCHI, Flawil, Switzerland) to concentrate the extract.

### 3.4. Determination of GABA Content in GBR and BR

GABA content in GBR and BR was determined using Liquid Chromatograph-Mass Spectrometer (LCMS) (Waters 2695, Wilford, MA, USA). Separation was carried out on C18 column (250 cm × 4.6 cm × 5 μm). Buffer A (0.1 M ammonium acetate; for 50 min) and buffer B (0.1 M ammonium acetate: acetonitrile: methanol) (44:46:10) were used as the mobile phases. Flow rate was maintained at 1.0 mL/min.

For analysis, GBR and BR extracts (50 μL each) were aliquoted into test tubes predried under vacuum. A 20 μL mixture of methanol: water: triethylamine (TEA) (2:2:1) was then added to these tubes, mixed well and dried under vacuum. Phenyl isothiocyanate (PITC) reagent (30 μL) composed of methanol, PITC, TEA and water mixed in a ratio of 7:1:1:1 was added to these tubes and contents were again dried under vacuum. Ammonium acetate buffer (500 μL; 0.1 M) was then added to each tube and resulting mixture was filtered into the vials using 0.45 μm syringe filter.

### 3.5. Cell Culture

The human neuroblastoma SH-SY5Y cells were grown in complete culture medium containing mixture (1:1) of Minimum essential Eagle’s medium and Ham’s nutrient mixture F-12, which was supplemented with 10% fetal bovine serum, 1% MEM non-essential amino acids and 50 μg/mL gentamicin. Cells were maintained at 37 °C under 5% CO_2_/95% air. Dimethyl sulfoxide (DMSO) concentration was maintained at 0.1% for all cell culture assays.

### 3.6. MTT Assay

The ability of GBR, BR and GABA to protect SH-SY5Y cells from H_2_O_2_ was determined by MTT assay, which is a potential indicator of cells viability. SH-SY5Y cells were seeded into 96-well culture plates at density of 1 × 10^5^ cells/mL and were allowed to attach. After 24 h, cells were differentiated with retinoic acid (10 μM) for 6 days prior to treatment. To examine the possible toxic effects, the cells were treated with GBR, BR and GABA individually over a concentration range of 0.25–200 μg/mL for 24 h. Similarly, cells were treated with H_2_O_2_ over concentration range of 50–400 μM up to 24 h. For the determination of neuroprotective effects, cells were pretreated with GBR, BR and GABA diluted in serum-free medium for 24 h and then challenged with H_2_O_2_ for another 24 h. GBR, BR and GABA were dissolved in DMSO which was maintained at 0.1% as this concentration showed no toxicity to the cells. MTT (Sigma, St. Louis, MO, USA) was added to all the wells and allowed to incubate in dark at 37 °C for 4 h. The amount of MTT formazan product was determined by measuring absorbance at 540 nm using a Microplate reader (Opsys MR, Thermo Labsystems, Franklin, MA, USA). All the MTT assays were performed in triplicate.

### 3.7. Cell Cycle Analyses

SH-SY5Y cells were seeded in 96-well plates at density of 1 × 10^5^ cells/mL. The cells were differentiated with 10 μM retinoic acid after 6 days. The cells were pretreated with each of the GBR, BR and GABA individually at 100 μg/mL for 24 h before exposing to 250 μM H_2_O_2_ for another 24 h. The cells were harvested using 0.1% trypsin-EDTA, fixed in 70% ethanol and kept at −20 °C overnight. After fixation, the pellets were washed with PBS to remove ethanol and mixed with 25 μL of RNAs, 50 μL of propidium iodide and 425 μL of PBS to make up the volume up to 500 μL. After 30 min of incubation in the dark, the DNA contents of the cells were analyzed using flow cytometer with Summit v4.3 software (Cyan ADP, Beckman Coulter, Brea, CA, USA).

### 3.8. Measurement of Mitochondrial Membrane Potential (MMP)

SH-SY5Y cells were seeded in 96-well plates at density of 1 × 10^5^ cells/mL. The cells were differentiated with 10 μM retinoic acid for 6 days and then pretreated with GBR and GABA at 10, 50 and 100 μg/mL for 24 h before being exposed to 250 μM H_2_O_2_ for another 24 h. Mitochondrial membrane was monitored using the fluorescent dye Rhodamine 123, which preferentially partitions active mitochondria based on the highly negative MMP. Depolarization of MMP results in the loss of Rhodamine 123 from mitochondria and a decrease in intracellular fluorescence is observed. Rhodamine 123 (final concentration of 10 μM) was added to the harvested cells and analyzed using flow cytometer with Summit software (Version 4.3; CyAN ADP, Beckman Coulter: Brea, CA, USA).

### 3.9. Annexin V-FITC and Propidium Iodide Staining Assay

To detect the effects of GBR, BR and GABA on early and late apoptosis/necrosis induced by H_2_O_2_, SH-SY5Y cells were stained with FITC-conjugated Annexin V and propidium iodide. SH-SY5Y cells were seeded in 96-well plates at density of 1 × 10^5^ cells/mL. The cells were differentiated with 10 μM retinoic acid for 6 days and pretreated with GBR at 1, 50 and 100 μg/mL for 24 h before being exposed to 250 μM H_2_O_2_ for another 24 h. The cells were then harvested using 0.1% trypsin-EDTA and cell pellets were resuspended in ice-cold 1X binding buffer. One microliter of Annexin V-FITC solution and 5 μL of propidium iodide were added to 100 μL of cells suspension. The tube was incubated on ice for 15 min in the dark followed by addition of 400 μL ice-cold 1X binding buffer and mixing gently. The samples were analyzed using flow cytometer with Summit software (Version 4.3; CyAN ADP, Beckman Coulter: Brea, CA, USA).

### 3.10. DPPH Radical Scavenging Assay

Stock solutions of GBR, BR and GABA were prepared as 5 mg/mL and dissolved in DMSO. Fifty microlitres of individual solutions were pipetted into a 96-well microtitre plate and mixed with 195 μL of 0.1 mM DPPH methanolic solution. The plate was swirled gently for 1 min and incubated in the dark at room temperature up to 60 min. Absorbance was read at 540 nm using Microplate reader (Opsys MR, Thermo Labsystem, Franklin, MA, USA). Trolox was used as standard and analyses were carried out in triplicate. Percent inhibition of DPPH radical was calculated using the following formula:

$$\text{Percent inhibition }(\text{I }\%)=\frac{{\text{Abs}}_{\text{Control }(\text{DPPH})}-{\text{Abs}}_{\text{Sample }\;(\text{DPPH}+\text{Sample})}}{{\text{Abs}}_{\text{Control}}}\times 100$$

DPPH scavenging activities of GBR, BR and GABA were expressed as mg Trolox equivalent per gram of extract (mg TE/g extract).

### 3.11. ABTS Radical Cation Scavenging Assay

ABTS radical cation was generated through oxidation of 7 mM ABTS with 2.45 mM potassium persulfate and incubated overnight in the dark at room temperature. ABTS radical cation solution was then diluted with ethanol to obtain an absorbance of 0.70 ± 0.02 at 734 nm spectrophotometerically (Pharmaspec UV-1700, Shimadzu, Kyoto, Japan). GBR, BR and GABA (50 μL each) were individually mixed with 950 μL of diluted ABTS solution and incubated for 10 min at room temperature. The absorbance was measured again at 734 nm. All the determinations were carried out in triplicate and the readings were averaged. Trolox was used as standard and percentage of ABTS radical cation decolorization inhibition was calculated using the following formula:

$$\text{Percent of inhibition }(\text{I }\%)=\frac{{\text{Abs}}_{\text{Control }(\text{ABTS})}-{\text{Abs}}_{\text{Sample }\;(\text{ABTS}+\text{Sample})}}{{\text{Abs}}_{\text{Control}}}\times 100$$

ABTS radical cation scavenging activity of GBR, BR and GABA was expressed in mg Trolox equivalent per gram extracts (mg TE/g extract).

### 3.12. Ferrous Ion Chelating Ability Assay

GBR, BR and GABA (1 mL each) was mixed with FeCl_2_ (0.1 mL; 2 mM) and ferrozine (50 μL; 5 mM) solutions. The absorbance at 562 nm was determined spectrophotometerically (Pharmaspec UV-1700, Shimadzu, Kyoto, Japan) 10 min after mixing the contents. The ferrous ion chelating ability was calculated as follows:

$$1-\frac{\text{Abs}.\text{sample}}{\text{Abs}.\text{control}}\times 100$$

The ferrous ion chelating ability of GBR, BR and GABA was expressed as mg EDTA equivalent per g extract (mg EDTA eq/g extract).

### 3.13. Statistical Analysis

Statistical analysis was conducted by one-way ANOVA, Tukey’s multiple comparisons and Student’s *t*-test using Statistical Package for Social Science (SPSS) version 20. *p* < 0.05 was considered as statistically significant difference.

## 4. Conclusions

In conclusion, the present study demonstrated that GBR is able to ameliorate H_2_O_2_-induced cytotoxicity and cell death in SH-SY5Y cells. The neuroprotective effects of GBR may be related to its antioxidant activity as GBR prevented cellular toxicity, and cell death by inhibiting cell cycle G0/G1 arrest for DNA damage repair. GBR also inhibited early and late apoptosis and restored collapse of MMP in response to H_2_O_2_ treatment. Further studies will be needed to investigate the mechanism of action for the protective effects of GBR.

## Figures and Tables

**Table 1:** 2,2-Diphenyl-1-picrylhydrazyl (DPPH) radical, 2,2′-azino-bis(3-ethylbenzothiazoline-6-sulphonic acid) (ABTS) radical cation and ferrous ion chelating potential of GBR, BR and GABA.

Samples	GABA content (μg/mL)	DPPH (mg TE/g extract)	ABTS (mg TE/g extract)	Ferrous ion chelating (mg EDTAE/g extract)
GBR	50 ± 1.22 ^a^	5.66 ± 0.04 ^a^	13.97 ± 0.12 ^a^	4.55 ± 0.09 ^a^
BR	9 ± 0.54 ^b^	4.32 ± 0.39 ^b^	11.82 ± 0.50 ^b^	4.26 ± 0.24 ^a^
GABA	NA	1.77 ± 0.46 ^c^	0.96 ± 0.29 ^c^	0.27 ± 0.18 ^b^

TE: Trolox equivalents, EDTAE: EDTA equivalents, NA: not applicable; Results are the mean ± SD (*n* = 3). Values with different superscripts in the same column are significantly difference, *p* < 0.05.

## References

[1] Ruffels J., Griffin M., Dickenson J.M. (2004). Activation of ERK1/2, JNK and PKB by hydrogen peroxide in human SH-SY5Y neuroblastoma cells: Role of ERK1/2 in H_2_O_2_-induced cell death. Eur. J. Clin. Pharmacol.

[2] Fordel E., Thijs L., Martinet W., Lenjou M., Lauf T., Bockstaele D.V., Moens L., Dewild S. (2006). Neuroglobin and cytoglobin overexpression protects human SH-SY5Y neuroblastoma cells against oxidative stress-induced cell death. Neurosci. Lett.

[3] Kwon S.H., Kim J.A., Hong S.I., Jung Y.H., Kim H.C., Lee S.Y., Jang C.G. (2011). Loganin protects against hydrogen peroxide-induced apoptosis by inhibiting phosphorylation of JNK, p38, and ERK 1/2 MAPKs in SH-SY5Y cells. Neurochem. Int.

[4] King K.L., Cidlowski J.A. (1995). Cell cycle and apoptosis: Common pathways to life and death. J. Cell Biochem.

[5] Sherr C.J. (1994). G1 phase progression: Cycling on cue. Cell.

[6] Finney P.L. (1978). Potential for the Use of Germinated Wheat and Soybeans to Enhance Human Nutrition. Advances in Experimental Medicine and Biology.

[7] Tkachuk R. (1979). Free amino acids in germinated wheat. J. Sci. Food Agric.

[8] Saikusa T., Horino T., Mori Y. (1994). Distribution of free amino acids in the rice kernel and kernel fractions and the effect of water soaking on the distribution. J. Agric. Food Chem.

[9] Kayahara H., Tsukahara K Flavor, Health and Nutritional Quality of Pre-Spruted Brown Rice.

[10] Tian S., Nakamura K., Kayahara H. (2004). Analysis of phenolic compounds in white rice, brown rice and germinated brown rice. J. Agric. Food Chem.

[11] Komatsuzaki N., Tsukahara K., Toyoshima H., Suzuki T., Shimizu N., Kimura T. (2007). Effect of soaking and gaseous treatment on GABA content in germinated brown rice. J. Food Eng.

[12] Usuki S., Ito Y., Morikawa K., Kise M., Ariga T., Rivner M., Yu R.K. (2007). Effect of pre-germinated brown rice intake on diabetic neuropathy in streptozotocin-induced diabetic rats. Nutr. Metab.

[13] Esa N.M., Kadir K.K.A., Amom Z., Azlan A. (2011). Improving the lipid profile in hypercholesterolemia-induced rabbit by supplementation of germinated brown rice. J. Agric. Food Chem.

[14] Norazalina S., Norhaizan M.E., Hairuszah I., Nurul-Husna S. (2011). Optimization of optimum condition for phytic acid extraction from rice bran. Afr. J. Plant Sci.

[15] Zhang R., Lu H., Tian S., Yin J., Chen Q., Ma L., Cui S., Niu Y. (2010). Protective effects of pre-germinated brown rice diet on low levels of Pb-induced learning and memory deficits in developing rat. Chem. Biol. Interact.

[16] Mamiya T, Kise M., Morikawa K., Aoto H., Ukai M., Noda Y. (2007). Effects of pre-germinated brown rice on depression-like behavior in mice. Pharmacol. Biochem. Behav..

[17] Mamiya T., Asanuma T., Kise M., Ito Y., Mizukuchi A., Aoto H., Ukai M. (2004). Effects of pre-germinated brown rice on β-amyloid protein-induced learning and memory deficits in mice. Biol. Pharm. Bull..

[18] Soi-Ampornkul R., Junnu S., Kanyok S., Liammongkolkul S., Katanyoo W., Umpornsirirat S. (2012). Antioxidative and neuroprotective activities of the pre-germinated brown rice extract. Food Nutr. Sci.

[19] Jung C.H., Hong M.H., Kim J.H., Lee J.Y., Ko S.G., Cho K., Seog H.M. (2007). Protective effect of a phenolic-rich fraction from *Schisandra chinensis* against H_2_O_2_-induced apoptosis in SH-SY5Y cells. J. Pharm. Pharmacol.

[20] Olivieri G., Otten U., Meier F., Baysang G., Dimitriades-Schmutz B., Muller-Spahn F., Savaskan E. (2003). Amyloid modulates tyrosine kinase B receptor expression in SHSY5Y neuroblastoma cells: Influence of the antioxidant melatonin. Neuroscience.

[21] Ismail N., Ismail M., Latiff L., Mazlan M., Mariod A. (2008). Black cumin seed (*Nigella sativa* Linn.) oil and its fractions protect against beta amyloid peptide-induced toxicity in primary cerebellar granule neurons. J. Food Lipids.

[22] Jeffrey A.K., Susan L.A. (2003). Oxidative stress, cell cycle, and neurodegeneration. J. Clin. Invest.

[23] Kruman I.I. (2004). Why do neurons enter the cell cycle?. Cell Cycle.

[24] Folch J., Junyent F., Verdaguer E., Auladell C., Pizarro J.G., Beas-Zarate C., Pallàs M., Camins A (2011). Role of cell cycle re-rntry in neurons: A common apoptotic mechanism of neuronal cell death. Neurotox. Res.

[25] Schwartz E.I., Smilenov L.B., Price M.A., Osredkar T., Baker R.A., Ghosh S., Shi F.D., Vollmer T.L., Lencinas A., Stearns D.M. (2007). Cell cycle activation in postmitotic neurons is essential for DNA repair. Cell Cycle.

[26] Wang W., Sun F., An Y., Ai H., Zhang L., Huang W., Li L. (2009). Morroniside protects human neuroblastoma SH-SY5Y cells against hydrogen peroxide-induced cytotoxicity. Eur. J. Pharmacol.

[27] Wang W., Huang W.T., Li L., Ai H.X., Sun F.L., Liu C., An Y. (2008). Morroniside prevents peroxide-induced apoptosis by induction of endogenous glutathione in human neuroblastoma cells. Cell Mol. Neurobiol.

[28] Wettasinghe M., Shahidi F. (2000). Scavenging of reactive-oxygen species and DPPH free radicals by extracts of borage and evening primrose meals. Food Chem..

[29] Netzel M., Strass G., Bitsch I., Könitz R., Christmann M., Bitsch R. (2003). Effect of grape processing on selected antioxidant phenolics in red wine. J. Food Eng.

[30] Miller D.M. (1996). Minerals.

[31] Thompson M., Williams C.R. (1976). Stability of flavonoid complexes of copper (II) and flavonoid antioxidant activity. Anal. Chim. Acta.

[32] Tian S., Nakamura K., Kayahara H. (2004). Analysis of phenolic compounds in white rice, brown rice and germinated brown rice. J. Agric. Food Chem.

[33] Xu Q., Kanthasamy A.G., Reddy M.B. (2011). Phytic acid protects against 6-hydroxydopamine-induced dopaminergic neuron apoptosis in normal and iron excess conditions in a cell culture model. Park. Dis.

[34] Mamiya T, Kise M, Morikawa K. (2008). Ferulic acid attenuated cognitive deficits and increase in carbonyl proteins induced by buthionine-sulfoximine in mice. Neurosci. Lett..

[35] Guizani N., Waly M.I., Ali A., Al-Saidi G., Singh V., Bhatt N., Rahman M.S. (2011). Papaya epicarp extract protects against hydrogen peroxide-induced oxidative stress in human SH-SY5Y neuronal cells. Exp. Bio. Med.

[36] Fukui K., Takatsu H., Koike T., Urano S. (2011). Hydrogen peroxide induces neurite degeneration: Prevention by tocotrienols. Free Radic. Res.

